# Supplementary material for: Liquid Crystal-Based Organosilicone Elastomers with Supreme Mechanical Adaptability
Source: Polymers (Basel). 2022 Feb 18;14(4):789. doi: 10.3390/polym14040789 (PMC8880581; doi:10.3390/polym14040789)
Supplement: Supplementary file 1 [file polymers-14-00789-s001.zip › polymers-1556043-supplementary.pdf]

Liquid Crystal-based Organosilicon Elastomers with Supreme Mechanical Strength

Zhe Liu[a], Yuqi Xiong[b], Jinghao Hao[a], Hao Zhang[a], Xiao Cheng[a], Hua Wang[a], Wei Chen*[b], Chuanjian Zhou*[a]

[a] School of Materials Science and Engineering, Shandong University, Jinan 250061, China.
[b] National Synchrotron Radiation Lab and Anhui Provincial Engineering Laboratory of Advanced Functional Polymer Film, CAS Key Laboratory of Soft Matter Chemistry, University of Science and Technology of China, Hefei 230026, China
* Correspondence: wc003@ustc.edu.cn (Wei Chen) and zhouchuanjian@sdu.edu.cn (Chuanjian Zhou)

Supporting Information

Table S1. Summary of the composition and characterization of silicone oils with different vinyl content.

Sample	Polydimethylsiloxane (g)	D_4^{vi} (g)	M_v	Apparent viscosity (mPa.s，25°C)
a	300	0	51889[a]	10339 [b]
b	300	0.81	52692[a]	10398 [b]
c	300	3.68	48881[a]	9862 [b]

[a]Determined by ubbelodhe viscometer; [b]Determined by rheometer.

Table S2. Summary of 5%, 10%, maximum weight loss temperature and 800°C residual weight.

No.	5%weight loss temperature (°C)	10%weight loss temperature (°C)	Maximum weight loss temperature (°C)	800°C residual weight (%)
Sample 1	425	450	591	45
Sample 2	404	448	605	47
Sample 3	397	440	605	46
Sample 4	389	433	629	46
Sample 5	380	423	628	43
Sample 6	397	432	625	43

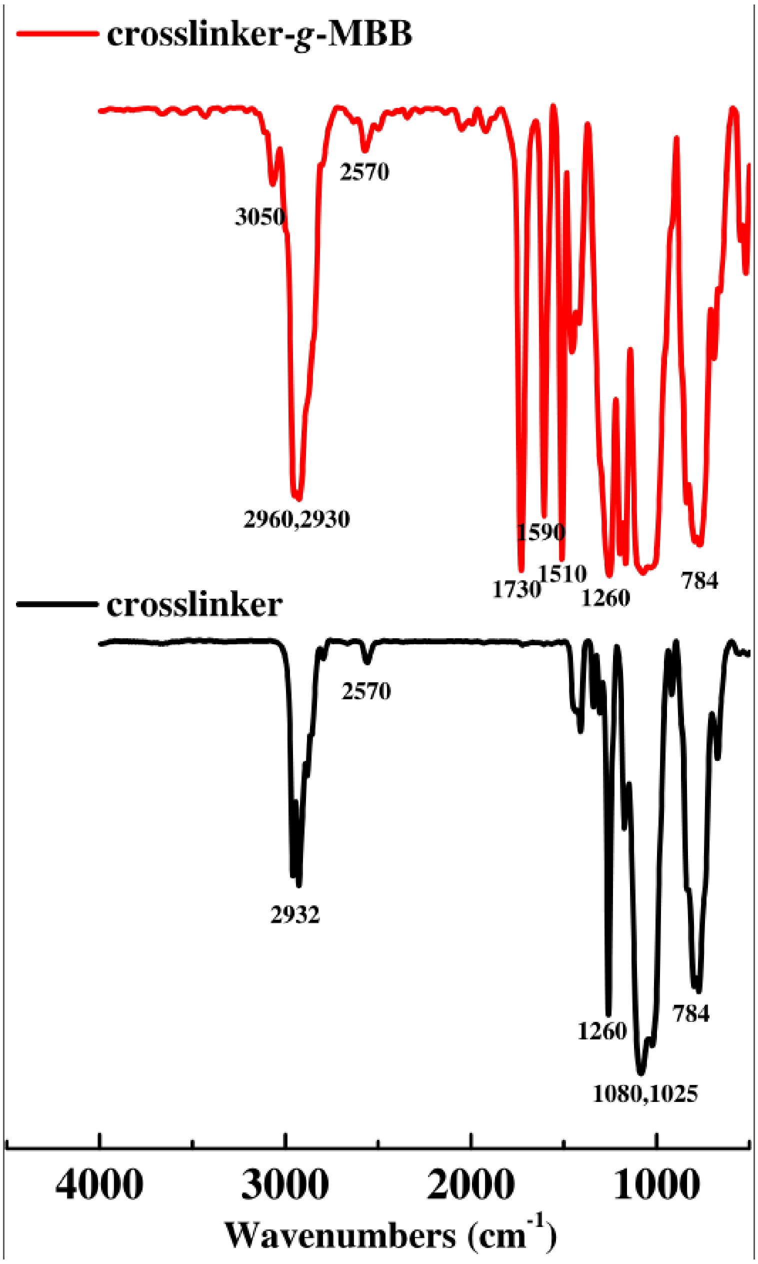

Figure S1. FT-IR spectra of crosslinker and crosslinker-*g*-MBB.

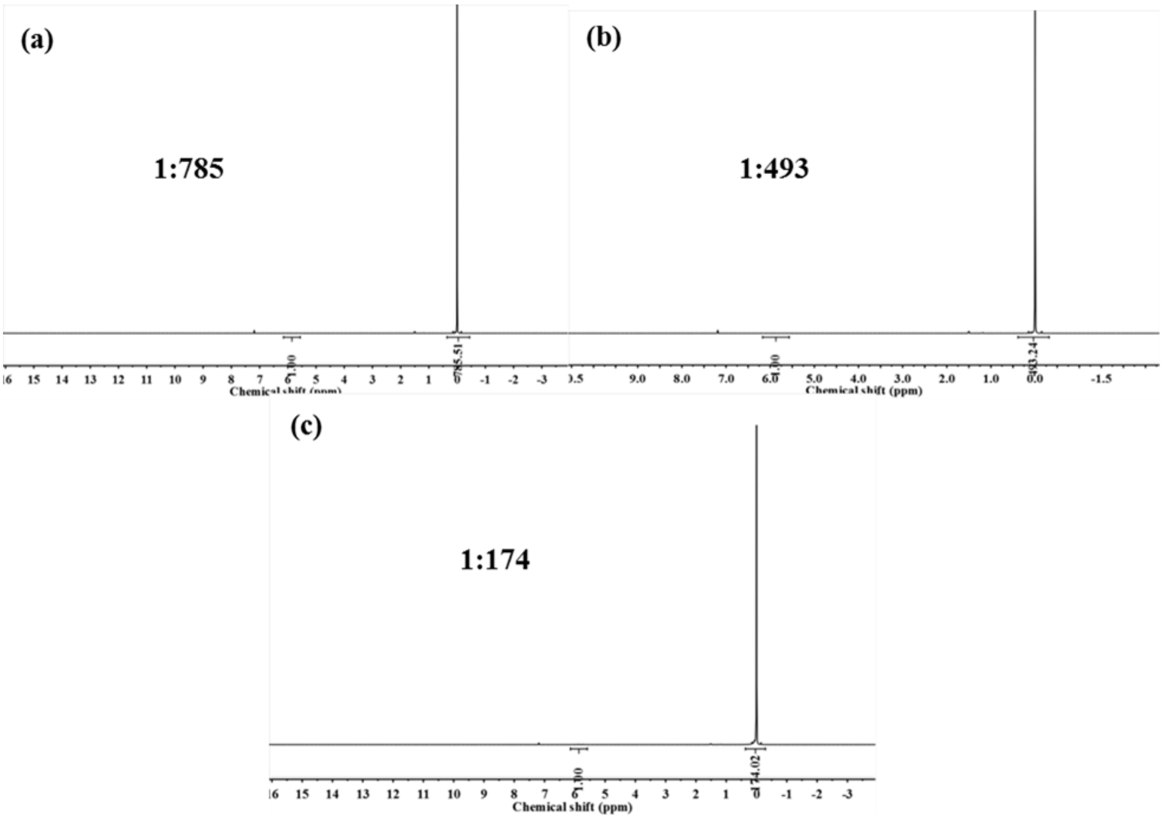

Figure S2. ^{1}H NMR spectra of three kinds of silicone oils with different vinyl content (a) Silicone oil (1‰ vinyl); (b) Silicone oil (2‰ vinyl); (c) Silicone oil (5‰ vinyl).

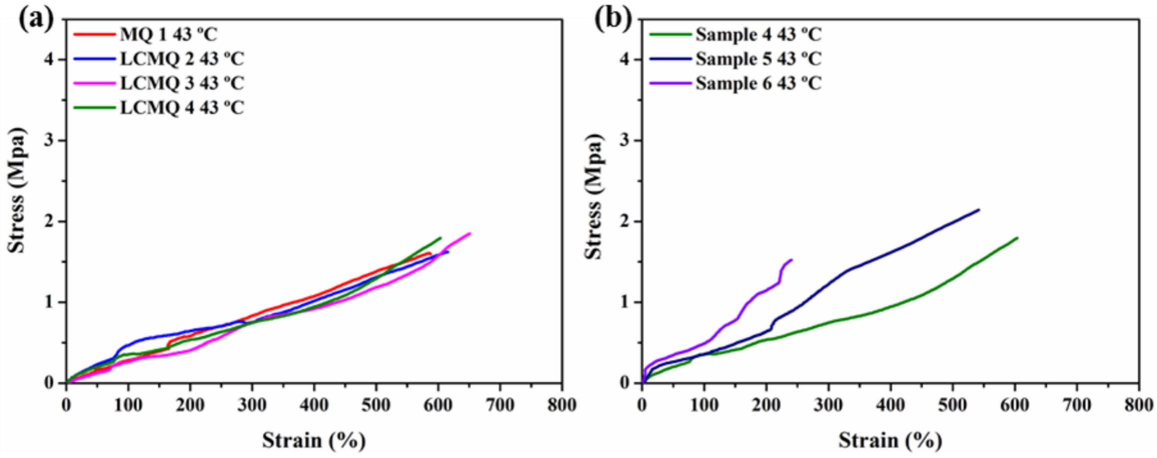

Figure S3. Stress-strain curves of (a) MQ 1 and LCMQs 2–4 and (b) LCMQs 4–6. The curves were performed at 43 °C.

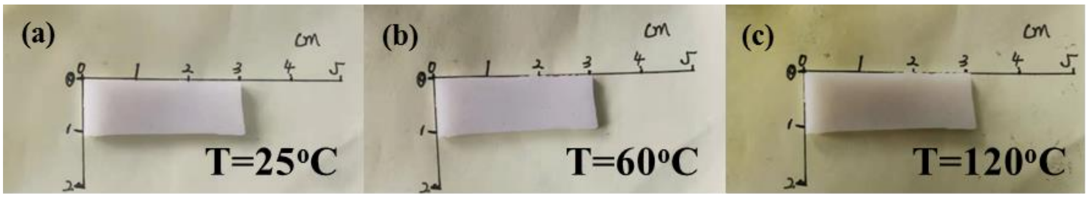

Figure S4. The images of (a) LCMQ 4 at 25°C; (b) LCMQ 4 at 60°C; (c) LCMQ 4 120°C.

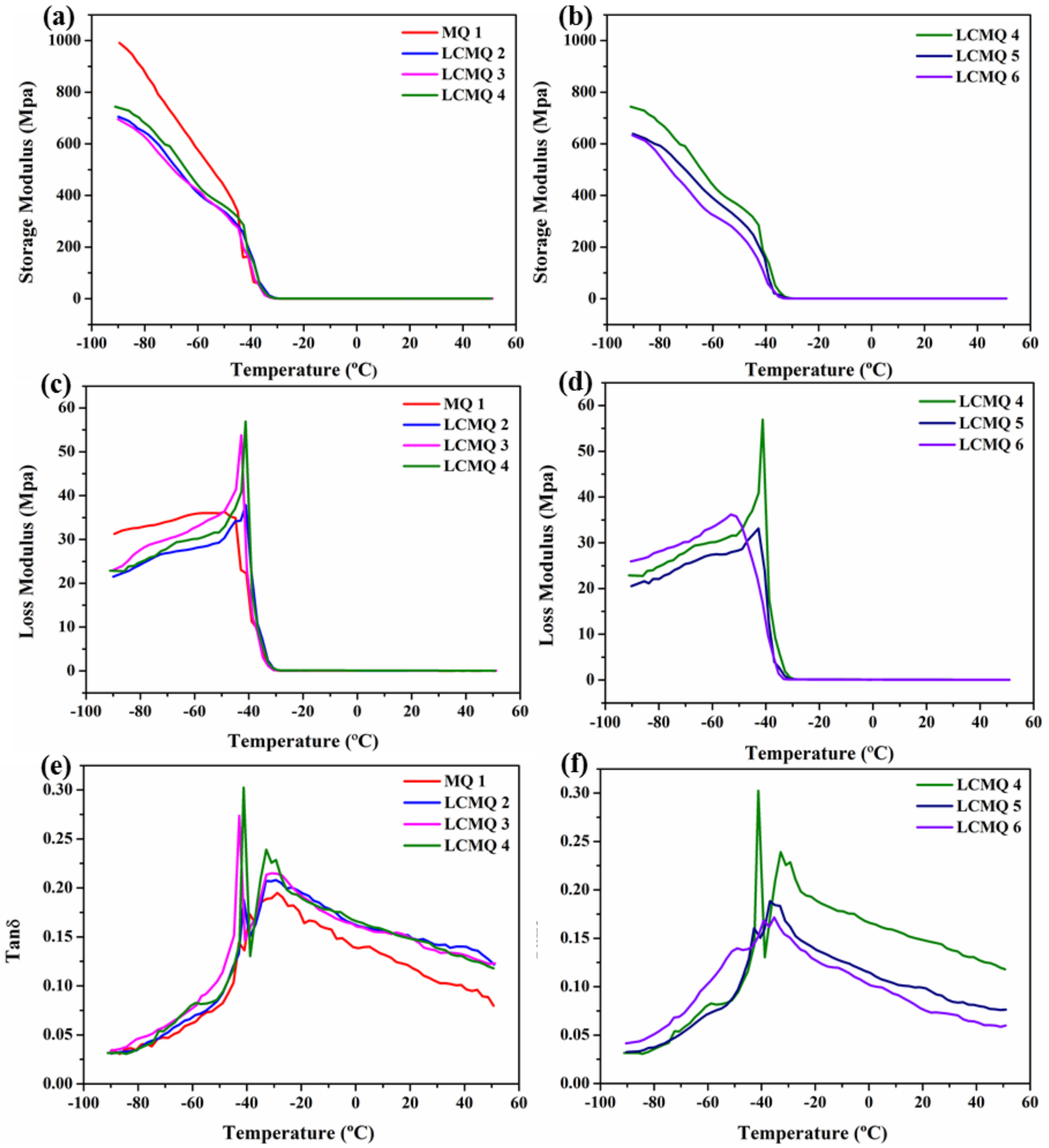

Figure S5. Storage modulus curves of (a) MQ 1–4 and (b) LCMQ 4–6**;** loss modulus curves of (c) MQ 1–4 and (d) LCMQ 4–6; loss factor curves of (e) MQ 1–4 and (f) LCMQ 4–6.
